# Resection-inspired histopathological diagnosis of cerebral cavernous malformations using quantitative multiphoton microscopy

**DOI:** 10.7150/thno.77532

**Published:** 2022-09-11

**Authors:** Shu Wang, Yueying Li, Yixuan Xu, Shiwei Song, Ruolan Lin, Shuoyu Xu, Xingxin Huang, Limei Zheng, Chengcong Hu, Xinquan Sun, Feng Huang, Xingfu Wang, Jianxin Chen

**Affiliations:** 1 College of Mechanical Engineering and Automation, Fuzhou University, Fuzhou 350108, China.; 2 Key Laboratory of OptoElectronic Science and Technology for Medicine of Ministry of Education, Fujian Provincial Key Laboratory of Photonics Technology, Fujian Normal University, Fuzhou 350007, China.; 3 Department of Neurosurgery, Fujian Medical University Union Hospital, Fuzhou 350001, China.; 4 Department of Radiology, Fujian Medical University Union Hospital, Fuzhou 350001, China.; 5 Department of General Surgery, Nanfang Hospital, Southern Medical University, Guangzhou 510515, China.; 6 Department of Pathology, the First Affiliated Hospital of Fujian Medical University, Fuzhou 350004, China.

**Keywords:** Multiphoton microscopy, cerebral cavernous malformations, hemosiderin, blood vessel, deep learning.

## Abstract

**Rationale:** Cerebral cavernous malformation (CCM) is prone to recurring microhemorrhage, which can lead to drug-resistant epilepsy. Surgical resection is the first choice to control seizures for CCM-associated epilepsy. At present, removal of resection-related tissue only depends on cautious visual identification of CCM lesions and perilesional yellowish hemosiderin rim by the neurosurgeon. Inspired by the resection requirements, we proposed quantitative multiphoton microscopy (qMPM) for a histopathology-level diagnostic paradigm to assist clinicians in precisely complete resection.

**Methods:** A total of 35 sections specimens collected from 12 patients with the CCM-related epilepsy were included in this study. First, qMPM utilized a label-free multi-channel selective detection to image the histopathological features based on the spectral characteristics of CCM tissues. Then, qMPM developed three customized algorithms to provide quantitative information, a vascular patterns classifier based on linear support vector machine, visualization of microhemorrhage regions based on hemosiderin-related parameters, and the CCM-oriented virtual staining generative adversarial network (CCM-stainGAN) was constructed to generate two types of virtual staining.

**Results:** Focused on CCM lesion and perilesional regions, qMPM imaged malformed vascular patterns and micron-scale hemosiderin-related products. Four vascular patterns were automatically identified by the classifier with an area under the receiver operating characteristic curve of 0.97. Moreover, qMPM mapped different degrees of hemorrhage regions onto fresh tissue while providing three quantitative hemosiderin-related indicators. Besides, qMPM realized virtual staining by the CCM-stainGAN with 98.8% diagnostic accuracy of CCM histopathological features in blind analysis. Finally, we provided pathologists and surgeons with the qMPM-based CCM histopathological diagnostic guidelines for a more definitive intraoperative or postoperative diagnosis.

**Conclusions:** qMPM can provide decision-making supports for histopathological diagnosis, and resection guidance of CCM from the perspectives of high-resolution precision detection and automated quantitative assessment. Our work will promote the development of MPM diagnostic instruments and enable more optical diagnostic applications for epilepsy.

## Introduction

Cerebral cavernous malformation (CCM) is a congenital cerebrovascular disease characterized by recurrent microhemorrhage, which can lead to drug-resistant epilepsy 1-4. Within 5 years, the recurrence rate of epilepsy in CCM patients with new-onset seizures can reach 94% 5. Surgical resection is the first choice to control seizures for CCM associated with epilepsy. The CCM lesion and the surrounding hemosiderin are critical factors to be considered in determining the extent of resection 2, 3, 6-8. Neurosurgeons frequently rely on the naked eye to detect the yellowish hemosiderin rim in the resection. In fact, there are some low concentrations of hemosiderin that sporadically remains in the surrounding brain parenchyma. From an imaging standpoint, incomplete excision of hemosiderin may be a potential reason for postoperative epilepsy recurrence. Therefore, it is significant to develop a higher-resolution imaging method that can effectively visualize the resection-related histopathological features in CCM tissues.

Magnetic resonance imaging (MRI) has been currently recognized as the most specific and sensitive technique for the preoperative diagnosis of CCM. The lesions and surrounding hemosiderin rings typically show heterogeneous low signal in T2* weighted gradient echo sequences and magnetic sensitivity weighted imaging 9. Besides, through quantifying CCM vascular permeability and iron deposition, dynamic contrast-enhanced quantitative perfusion and magnetically sensitive quantitative imaging techniques can reflect disease progression 10. However, due to the spatial resolution limitations of MRI, intraoperative CCM examination must rely on the histopathological diagnosis to reflect the distribution of hemosiderin and other microstructures at the cellular level. Inevitably, current clinic histopathological techniques still have multiple workflows, such as complex protocols and exogenous labeling. Additionally, with the current shortage of pathologists and the increasing biopsy demand for various diseases, the traditional pathological diagnosis method has further increased the burden and responsibility of pathologists.

Advanced optical microscopy, such as light-sheet microscopy 11 and ultraviolet surface excitation microscopy 12, enable the rapid imaging of intact tissues at high-resolution, but their imaging specimens need fluorescent labeling or even tissue clearing. Photoacoustic microscopy 13 and optical coherence tomography 14 can achieve clinical *in vivo* images at higher penetration depth without tissue processing or staining. Computational diffraction tomography has also recovered macroscale cellular membrane structures, subcellular organelles, and microorganism tissues 15. However, the single-channel images acquired by these techniques often combine physical model-based algorithms. Femtosecond pump-probe microscopy has been applied to differentiate hemoglobin and hemosiderin 16. Remarkably, this optical system requires experienced optical engineers to adjust two femtosecond laser beams with overlap spatially and temporally, which may be inadequate for medical researchers to operate. Compared with the clinical imaging and optical histopathological methods, multiphoton microscopy (MPM), which is based on the second harmonic generation (SHG) and two-photon excited fluorescence (TPEF), has developed into a reliable and easy-to-use imaging instrument for in-depth clinical research 17-19. Label-free MPM can simultaneously excite several endogenous signals in various components of biological tissues and acquire multi-channel multi-color images at the histopathology-scale resolution, making it the most likely to implement non-destructive tomography in clinical medicine 20.

**Figure 1:**
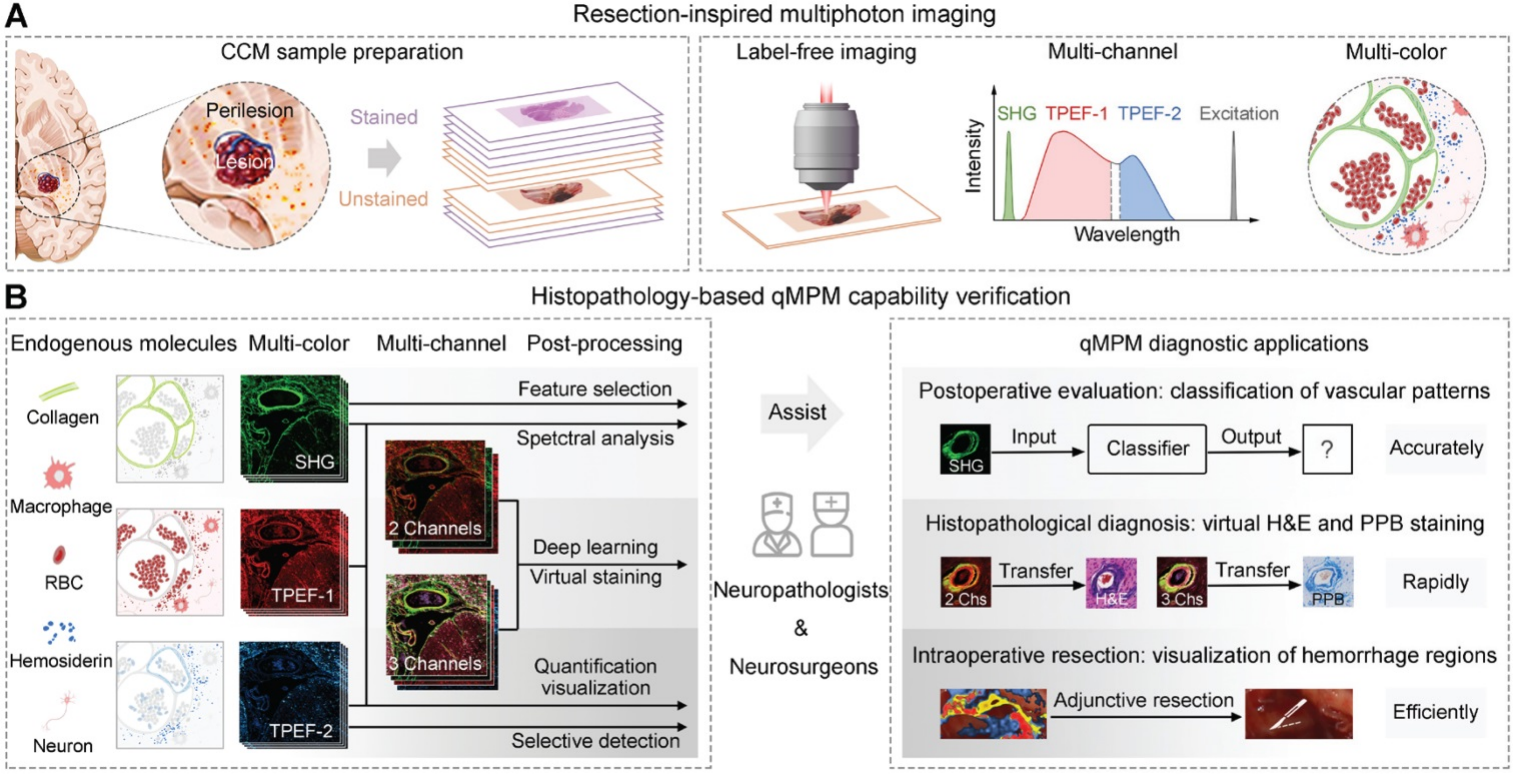
** Schematic diagram of qMPM and the capability for histopathology-based diagnostic applications. (A)** Inspired by the resection requirements, unstained CCM tissues were used for label-free imaging based on SHG, TPEF-1, and TPEF-2 channels to distinguish histopathological features with multicolor-coded. **(B)** Based on the principle of endogenous multiphoton imaging, qMPM has the capability to provide more accurate, rapid, and efficient clinical diagnostic guidance combined with custom-developed post-processing algorithms.

Here, we proposed quantitative MPM (qMPM), combined the front-end multi-channel selective detection modalities with the back-end multiple custom-developed image processing algorithms, and comprehensively verified the diagnostic capabilities of qMPM on CCM, inspired by the resection requirements. This work presented two main highlights (Figure 1). First, from the lesion to perilesional histopathological features, we utilized qMPM to excite multiple endogenous molecules based on the spectral characteristics, focused on the vascular patterns and the micron-scale hemosiderin-related products (Figure 1A). Second, from the optical imaging to clinical applications, qMPM realized three custom-developed algorithms, including a classifier of vascular patterns, a quantitative visualization of hemorrhage regions, and an unsupervised deep-learned model that can generate two types of virtual staining, assisting neuropathologists and neurosurgeons in making more precise CCM intraoperative resection guidance and histopathological diagnosis (Figure 1B).

## Results

### Multiphoton imaging and quantitative classification of vascular patterns in CCM lesion

**Figure 2:**
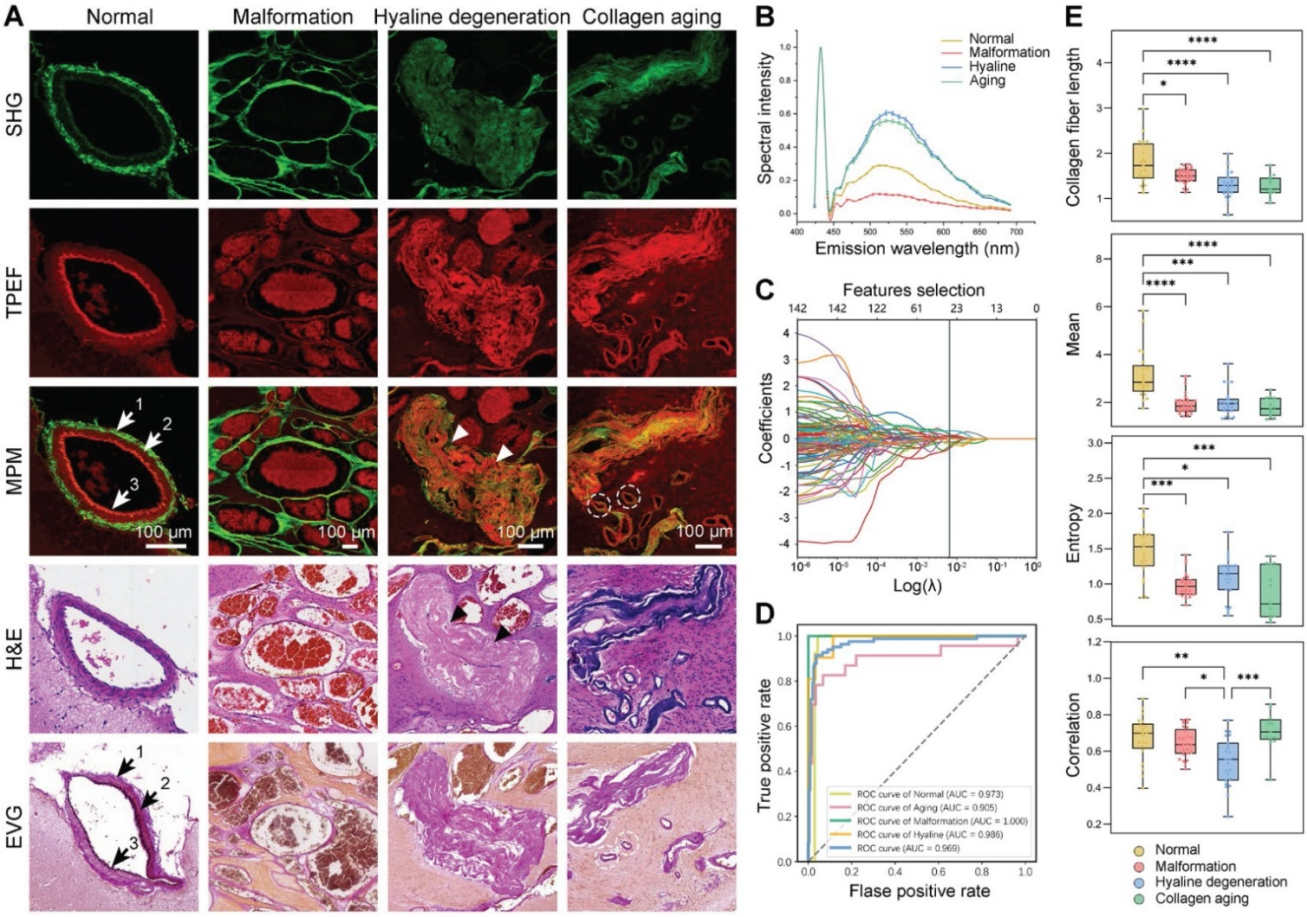
** qMPM can classify vascular patterns in CCM lesions. (A)** Representative vascular patterns with distinguishable features in MPM images. Normal vessels in overlaid images showed layered structures, collagen fiber (arrow 1), smooth muscle (arrow 2), and elastic fibers (arrow 3), corresponding to the black arrows in EVG staining images. Malformation vessels appeared as multiple dilated blood-filled caverns. Hyaline degeneration was homogeneous translucent without distinct vascular cavities (white arrowheads), similar to the black arrowheads in H&E staining images. Collagen aging was a vascular cavity with both SHG and TPEF signals (dashed circles). **(B)** The emission spectrum of different vascular patterns at an excitation wavelength of 860 nm. **(C)** The potential predictors were selected using LASSO logistic regression. **(D)** The ROC curves of the vascular pattern classifier were shown as the mean (n = 82). **(E)** The features selected by LASSO logistic regression and ANOVA statistical approach, shown as mean ± SEM (n = 82) in box plots. Centerlines, medians; limits, 75 and 25%; whiskers, maximum and minimum.

Histologically, the CCM lesions are characterized by a mulberry-like, multilobulated appearance within the brain parenchyma. To verify that qMPM imaging can identify vascular histopathological features, we first focused on the vascular patterns in the CCM lesions (Figure 2A). The morphologically normal vessels showed distinct layered structures, which were collagen fiber (arrow 1), smooth muscle (arrow 2), and elastic fibers (arrow 3). Collagen had a strong SHG signal due to its non-centrosymmetric structure. Both smooth muscle and elastic fiber showed TPEF signal. Elastic fibers presented the high TPEF signal and low SHG signal with a curled shape, which distinguished them from yellowish muscle fibers. Compared with normal blood vessel, we found three representative malformed vascular patterns (Figure 2A, the right three columns). Malformation vessels were comprised of multiple dilated blood-filled caverns with little or no intervening brain parenchyma, and they lacked the smooth muscle and elastic fiber observed in mature vascular vessels. Hyaline degeneration is the most common form of degeneration observed in CCM lesions. This degeneration was the formation of a homogeneous translucent zone in the vessel wall, displaying remarkable deposition of dense fibrillar collagen without an obvious vascular cavity (Figure 2A, arrowheads). The increasing dense SHG signal and intensified TPEF signal of hyaline can be distinguished from malformation vessels. Besides, we also found another type of twisted blood vessel with a vascular cavity (Figure 2A, dashed circles). The yellowish vessel wall displayed weak SHG and strong TPEF signals, which appeared blue in the corresponding hematoxylin-eosin (H&E) staining, indicating collagen aging vessels. The corresponding H&E staining confirmed the position and morphology of the vessels, while the Elastic van Gieson (EvG) staining highlighted blood vessel walls. The overlaid results were highly consistent with H&E and EvG stained images, indicating that qMPM could identify histopathological features of different vascular patterns in CCM lesions more efficiently than H&E staining. The slight differences primarily originated from the minor morphological shifts caused by tissue processing.

To quantitatively classify the vascular patterns in CCM lesions, we performed a spectral analysis of the vascular composition (Figure 2B). Collagen fibers showed a clear SHG signal at 430 nm. The positions of different vascular emission peaks were similar, which were mainly associated with nicotinamide adenine dinucleotide (NADH) (peak around 455 and 475 nm), elastin (peak around 511 nm), flavin adenine dinucleotide (FAD) (peak around 543 nm), and porphyrin derivatives (peak around 630 nm) 21. However, it was still difficult to distinguish between hyaline and aging vessels based on the spectral signatures and morphology. Therefore, to achieve more accurate vascular pattern classification, we extracted 142 spatial features of collagen fiber using collagen feature extraction algorithm (Table S1) 22, 23. Total 23 best potential predictors were selected from all features (Figure 2C). Subsequently, we developed the linear support vector machine (SVM) classifier using these 23 features to automatically classify vascular patterns, which showed performance with AUC value of 0.969 (Figure 2D). Besides, collagen fiber length, mean, entropy and correlation were jointly selected by LASSO logistic regression and ANOVA statistical approach, revealing the difference among the vascular patterns (Figure 2E). The significant correlation difference between hyaline and aging showed the potential to be utilized as indicators for vascular identification. These results suggested that qMPM has the ability to assist pathologists in automatically classifying and quantifying the four vascular patterns, which may reduce their workload and ensure adequate accuracy.

### Multiphoton imaging and spectral analysis of hemosiderin in CCM perilesional region

**Figure 3:**
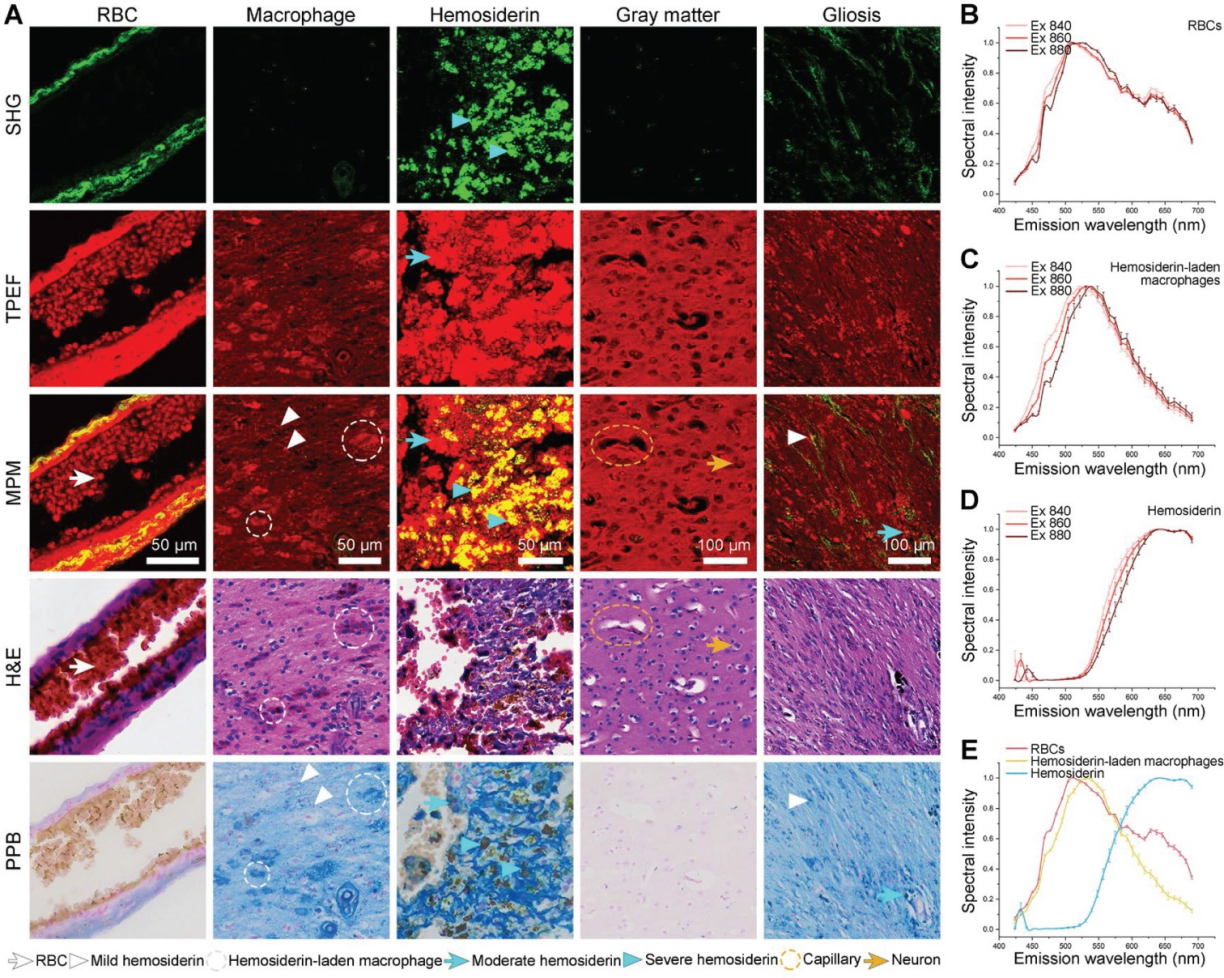
** qMPM can identify hemosiderin-related products in CCM perilesional region. (A)** MPM imaging of hemosiderin-related products. The red blood cells (RBCs), hemosiderin-laden macrophages, and hemosiderin represented the progression of blood breakdown products. **(B-D)** The spectral analysis of RBCs, hemosiderin-laden macrophages, and hemosiderin at three different excitation wavelengths. **(E)** The emission spectrum of three hemosiderin-related products.

Chronic deposition of blood breakdown products caused by recurrent microhemorrhages is the most critical feature in determining the extent of resection during CCM-associated epilepsy surgery. As a result, we proceeded to image the CCM perilesional regions that associated with resection. The red blood cells (RBCs), hemosiderin-laden macrophages, and hemosiderin have represented the progression of blood breakdown products (Figure 3A, the first three columns). The first stage was RBCs, which had an oval shape and were tightly packed in the blood vessels (Figure 3A, white arrows). Once RBCs are diffused into the brain tissue, they may be captured by macrophages, resulting in the production of hemosiderin-laden macrophages and further hemosiderosis. The phagocytosis of macrophages was involved in the degradation of hemoglobin and the formation of hemosiderin. Hemosiderin-laden macrophages were the second stage, which always presented in perilesional tissue and within the lesions. Hemosiderin-laden macrophages showed a foam-like shape and the nucleus of the dark hole surrounded by granular hemosiderin with a relatively strong TPEF signal (Figure 3A, white dashed circles). Besides, we can also capture specks of extracellular hemosiderosis (Figure 3A, white arrowheads). The third stage was hemosiderin. The rim of hemosiderin was frequently surrounded by CCM lesions, which was the significantly epileptogenic region due to iron deposition from recurrent hemorrhage. In the overlaid images, hemosiderin presented two intensity distributions. The first one showed red color, generating a strong TPEF signal without a SHG signal (Figure 3A, cyan arrows). The other showed yellow color, producing both strong SHG and TPEF signals (Figure 3A, cyan arrowheads). The corresponding Perls Prussian Blue (PPB) staining validated that these two intensity distributions were related to the different degrees of hemosiderosis, with only the hemorrhage center areas producing strong SHG signals. Capillaries and neurons showed orderly distribution with homogeneous TPEF signal in structurally normal gray matter (Figure 3A, yellow circles, and arrows). The last hallmark was gliosis, similar to hemosiderin, a potential epileptogenic feature caused by repetitive microhemorrhages. Gliosis had a higher cell density than gray matter due to focal proliferation, and it also had different degrees of hemosiderosis (Figure 3A, cyan arrows, and white arrowheads). In addition, the degenerated vessel wall exhibited a weak SHG signal due to collagen hyperplasia caused by the vascular lumen occlusion. qMPM, H&E, and PPB staining methods generated well-correlated images and validated qMPM revealed a similar distribution of perilesional features as the corresponding staining methods.

To quantitatively describe the progression of blood breakdown products, we conducted spectral analysis of RBCs, hemosiderin-laden macrophages, and hemosiderin at three different excitation wavelengths (Figure 3B-D). The signals of RBCs and hemosiderin-laden macrophages were mainly contributed by TPEF. The broad emission spectrum from RBCs had been assigned to several sources, including NADH (455 and 475 nm), hemoglobin (515 nm), FAD (543 nm), and porphyrins (585 nm and 630 nm) (Figure 3B) 24-26. RBCs were densely packed with large amounts of hemoglobin. The peaks at ~585 and 630 nm were due to two different forms of porphyrins released from RBCs 25. The spectrum was mostly emitted by cellular NADH and FAD in hemosiderin-laden macrophages (Figure 3C). In hemosiderosis, the emission spectrum had a minor SHG peak, and high-intensity broad TPEF peaks at 630 nm and 690 nm (Figure 3D). Because porphyrin molecules also existed in iron-binding complexes such as heme and ferritin containing hemosiderosis 21, 27, both porphyrins and iron complexes presented at higher concentrations in hemosiderin spectrum. As result, we considered that the TPEF peaks were primarily originated from porphyrin. Since most of the biophysical and biological properties of hemosiderin remain unknown, we hypothesized that the SHG signal might be generated by ferritin with a non-centrosymmetric structure in a condensed iron-protein complex. Finally, we compared the spectrum of three products at 860 nm excitation wavelength (Figure 3E). Spectral differences can be used to identify RBCs, hemosiderin-laden macrophages, and hemosiderin. In particular, the spectral intensity of hemosiderin was much stronger than the other two products at longer wavelengths (600-695 nm). The spectral and spatial differentiation of the three hemosiderin-related products not only demonstrated the interpretability of the imaging results, but also accurately characterized the hemorrhagic degree of CCM.

### Detection of CCM histopathological features in large-scale specimen by multichannel qMPM

**Figure 4:**
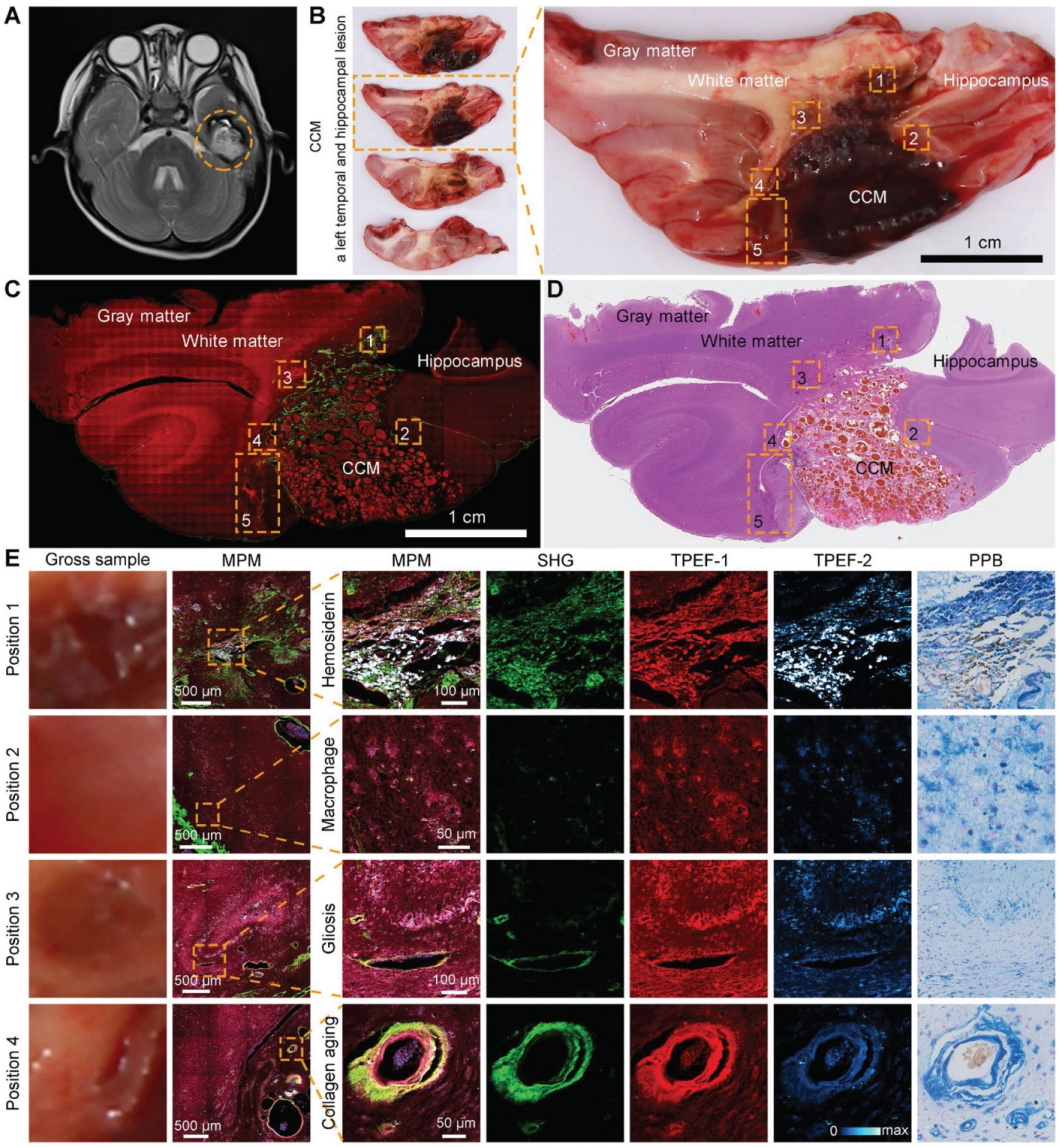
** qMPM can detect CCM histopathological features in the large-scale specimen. (A)** MRI of a left temporal CCM featured with a hypo-intense ring on the T2 sequence (dashed circle). **(B)** The corresponding fresh tissues including the CCM lesion and perilesional region. **(C, D)** Corresponding two-channel MPM image and H&E-stained image. **(E)** Perilesional areas of interest (dashed boxes) in the CCM gross sample (B) were captured for MPM imaging.

qMPM had an excellent histopathological-level correlation between the morphology of CCM in multiphoton and staining images. We performed qMPM on large-scale CCM specimens containing CCM lesions, perilesional regions, and structurally normal regions. An MRI of a left temporal CCM revealed hemosiderosis surrounding the lesion, which was emphasized by a hypo-intense ring on the T2 sequence (Figure 4A, dashed circle). The fresh tissues were removed from the gross margin of the lesion, including the CCM lesion and the surrounding areas. CCM was well-circumscribed lesions (Figure 4B). Hemosiderin rim appeared ambiguous yellowish color around the CCM lesions, which closely related to epilepsy. Therefore, intraoperative visualization of the CCM lesion and the hemosiderin in the CCM perilesional region is required to assist the neurosurgeons in the completely removing the surrounding hemosiderin.

**Figure 5:**
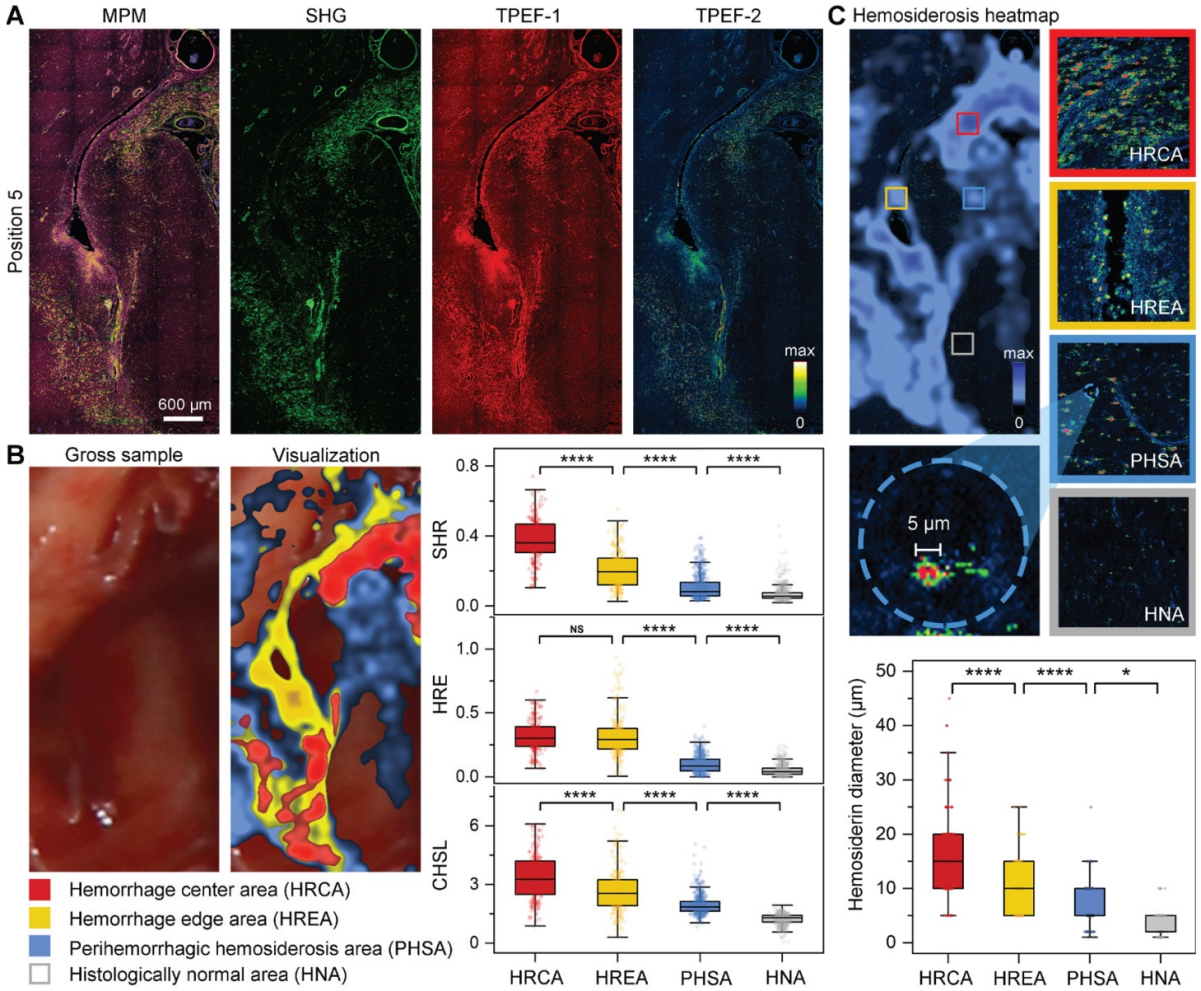
** qMPM can quantitatively visualize the hemorrhage-related areas. (A)** The enlarged image of the fifth position (dashed boxes in Figure 4B), with three-channel MPM and single-channel images respectively. Blue to yellow pseudo-color represents increased deposition intensity in the TPEF-2 channel, the strongest is red. **(B)** Quantitative visualization of hemorrhage-related areas and calculation of hemosiderin-related parameters (n*_HRCA_* = 211, n*_HREA_* = 245, n*_PHSA_* = 610, n*_HNA_* = 567, n depends on the size of the area, field of view (FOV) = 816 μm × 816 μm). SHR: serious hemorrhage ratio; HRE: hemorrhage extent; CHSL: cumulative hemosiderosis level. **(C)** The comparation of different degrees of hemosiderosis. The box plots quantitatively represent the hemosiderin diameter in these different deposition degrees (n*_HRCA_* = 152, n*_HREA_* = 90, n*_PHSA_* = 96, n*_HNA_* = 50, n depends on the quantity of hemosiderin, measured in twelve FOVs of 2041 μm × 2041 μm). Centerlines, medians; limits, 75 and 25%; whiskers, maximum and minimum.

We used two-channel MPM to grossly detect the microscopic morphology of the large-scale specimen. Gray matter, white matter, hippocampus, and CCM lesions can be clearly distinguished (Figure 4C). Inspired by the distinct spectral differences among hemosiderin-related products, we performed three-channel MPM to achieve higher contrast hemosiderin rim (Figure 4E). The first channel detected vascular collagen and severe hemosiderosis (SHG, 395 to 415 nm). The second channel was used to image cellular NADH and FAD, as well as blood vessel components other than collagen (TPEF-1, 428 to 570 nm). The third channel was mainly used to identify hemosiderosis (TPEF-2, 600 to 695 nm). We focused on five representative regions of interest in perilesional regions of the CCM gross sample (Figure 4B, dashed boxes). The enlarged images were presented in Figure 4B and Figure 5. The single-channel images can differentiate components based on spectral pseudo-color. The multi-color composite images can highlight the signal intensity and feature distribution. When the features appeared pink (Figure 4E), the signal of the TPEF-1 channel was relatively strong, indicating that the location was the vessel wall or the brain parenchyma contained NADH and FAD. If the image showed purple or even white, the signal of the TPEF-2 channel was stronger, implying that there was a certain amount of hemosiderosis at this location. In summary, we can select two-channel MPM to rapidly scan the large-scale specimen, or utilize three-channel MPM to more accurately diagnose CCM histopathological features and further perform quantitative histopathological analysis.

### Quantitative visualization of hemorrhage regions based on multichannel MPM images

Three-channel MPM images can precisely display the distribution of hemosiderin compared with the gross sample. Therefore, the fifth position selected the larger perilesional region containing hemosiderin at different deposition levels (Figure 5). There was obvious hemosiderosis near the blood vessels, as well as scattered hemosiderin deposition in the surrounding regions (Figure 5A). To enhance the applicability of qMPM in resection decisions, we quantitatively visualized the hemorrhage and hemosiderosis extent based on three hemosiderin-related parameters (i.e., serious hemorrhage ratio (SHR), hemorrhage extent (HRE), and cumulative hemosiderosis level (CHSL)). The SHR and HRE reflect different degrees of hemorrhage, respectively. The CHSL can reflect the accumulation of all hemosiderin, which is related to the pixel intensity and area of TPEF-2 channel. In Figure 5B, hemorrhage center area (HRCA, color-coded red), hemorrhage edge area (HREA, color-coded yellow), and perihemorrhagic hemosiderosis area (PHSA, color-coded blue) were visualized as a heatmap overlaid with gross sample. The uncovered area was considered to be the histologically normal area (HNA). The calculation of SHR, HRE, and CHSL in the four regions verified that the descent of parameter values was consistent with the distance from the hemorrhage center. Moreover, there were significant differences in the three hemosiderin-related parameters between perihemorrhagic hemosiderosis area and histologically normal area. Therefore, we believed that the blue perihemorrhagic hemosiderosis area could rapidly determine the extent of hemosiderosis. The visualization of the first position in the gross sample can be detailed in Figure S1.

In addition, qMPM can also select to acquire the multiphoton histopathological features in the visualized areas. Figure 5C qualitatively and quantitatively showed significant differences in hemosiderosis among the four areas. These perihemorrhagic hemosiderosis, like the infiltrating tumor cells, spread beyond the gross and radiographic margins, indicating that MPM can detect hemosiderin at various accumulation levels. Significantly, we found that 5 μm of hemosiderin deposits can often be detected in the perihemorrhagic hemosiderosis area, but rarely in the histologically normal area. As a result, qMPM provided a hemosiderin diameter-associated resection marker, that is, the location of the 5 μm hemosiderin could be used to precisely determine the hemosiderosis area. Therefore, combined the visualization areas, the diameters of hemosiderin deposits with the multichannel MPM images, qMPM could aid neurosurgeons in their analysis of the corresponding parameters, then provide a resection guidance for the yellowish hemosiderin rim and even a larger area.

### Evaluation of the histopathology-based qMPM diagnostic capability on CCM

**Figure 6:**
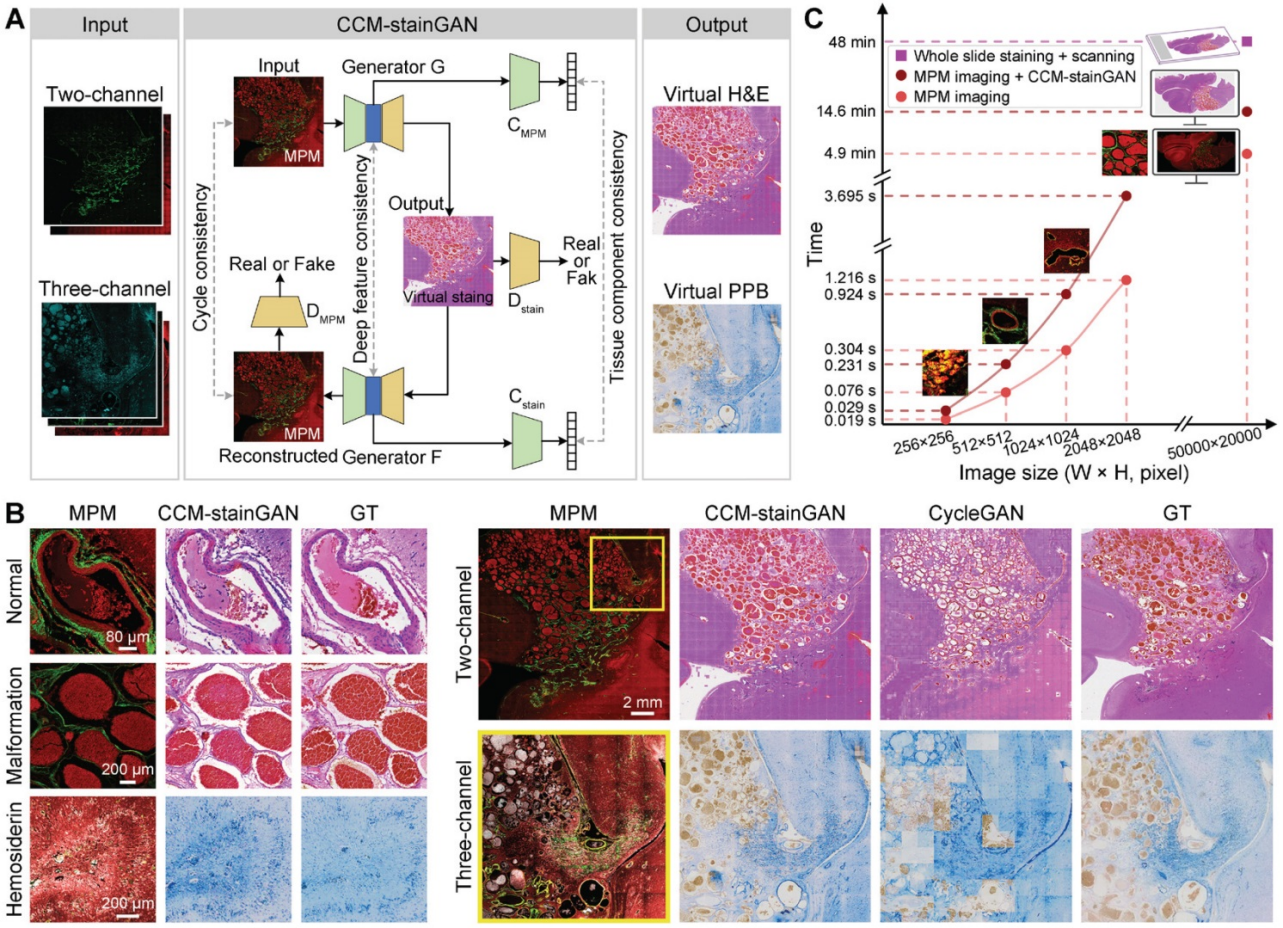
** qMPM images can be transformed into virtual stained images using CCM-stainGAN. (A)** The flowchart of CCM-stainGAN. Input MPM images are transformed into the virtual stained images by the forward generator (G), and then reconstructed to MPM images by the backward generator (F). Two classifiers (C_MPM_ and C_stain_) are built for inferring the category of tissue component. Two discriminators (D_MPM_ and D_stain_) are trained together with generators to evaluate the quality of transformed images and to improve the fidelity of generators. The cycle consistency constraint is enforced to guarantee the cycle-reconstructed MPM images as close to the input MPM images as possible. The deep feature consistency and the tissue component consistency ensure that the output virtual stained images have both the histopathological detailed features and the MPM image information. **(B)** The transformation results of typical CCM histopathological features. The images of adjacent stained sections were presented as ground truth (GT). Compared with CycleGAN, CCM-stainGAN preserved more histopathological structures and exhibited superior restorability in large-scale images. **(C)** Comparison of reference time consumption between multiphoton imaging combined with virtual staining and H&E digital scanning on images at different scales.

Although MPM can compensate for the shortcomings of traditional histopathological diagnosis, clinicians must be trained on MPM images before using them for auxiliary diagnosis. To tend the diagnostic capability of qMPM, we constructed an unsupervised deep learning model, the CCM-oriented virtual staining generative adversarial network (CCM-stainGAN), which can transform two-channel and three-channel MPM images into virtual H&E and PPB stained images respectively (Figure 6A). Typical CCM histopathological features such as normal vessels, vascular malformation and hemosiderosis were well preserved with high fidelity, accurately reconstructing an effect comparable to that of real H&E and PPB staining from MPM images (Figure 6B). To test the transformation of large-scale images, we partitioned them into multiple tiles and then stitched the predicted tiles together to obtain the final results. Compared to the CycleGAN, our model reconstructed richer and more realistic stained details from MPM images (Figure 6B). Notably, we compared the reference time consumed by MPM imaging combined with CCM-stainGAN and H&E digital scanning according to the scale of CCM histopathological features (Figure 6C). As a result, MPM imaging and virtual staining processes in qMPM can assist clinicians in more efficient diagnosis, which facilitated the adoption of qMPM in CCM histopathological diagnosis workflows.

**Figure 7:**
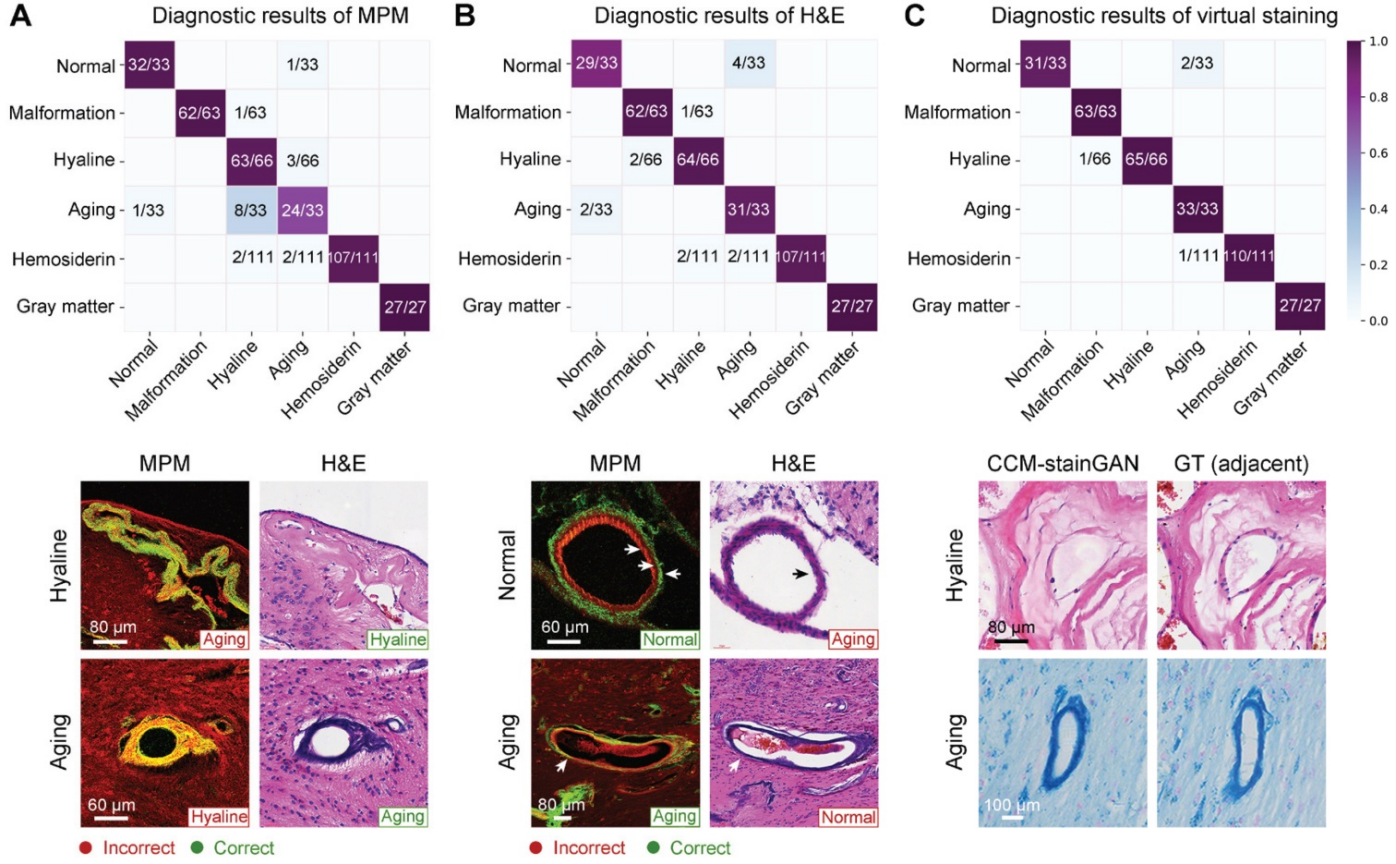
** qMPM implements with virtual staining have the capacity to assist neuropathologists in diagnosis. (A)** Confusion matrix of MPM diagnosis. Errors occurred mainly in mistaking aging for hyaline (8/33) and mistaking hyaline for aging (3/66). **(B)** Confusion matrix of H&E diagnosis. Mistakes occurred mainly between normal and aging vessels, with error rates of 4/33 and 2/33. **(C)** Diagnostic results of virtual staining. Reconstructed details of hyaline and aging vessels were improved compared with MPM. Besides, the normal vessels were correctly diagnosed in 31/33 due to the CCM-stainGAN preserving the histological structures in MPM images.

Subsequently, we performed a blind diagnostic analysis of MPM, virtual-stained, and H&E-stained images on three pathologists to verify the legibility of MPM and virtual-stained images (Table S2). The diagnostic accuracies of MPM were comparable to H&E for vascular malformation, hyaline degeneration, hemosiderin, and gray matter. The representative error types were shown in Figure 7. MPM images can distinguish between normal and aging vessels more effectively than H&E staining (Figure 7A), instead of hyaline and aging vessels with resembled morphology. While H&E-stained images might be beneficial for pathologists in distinguishing between aging and hyaline vessels (Figure 7B). In contrast, virtual staining, including H&E and PPB, performed the best diagnostic accuracy without requiring MPM training for pathologists (Table S2). The qMPM that MPM combined with CCM-stainGAN not only inherited the advantages of MPM but compensated for the deficiencies by allowing the unique characteristics of hyaline and aging vessels to be recreated realistically (Figure 7C).

Finally, we clarified the MPM histopathological diagnostic criteria in CCM by comparing it to their H&E histopathology (Table 1). The results showed that these two modal images were consistent in identifying the vascular structure, but the MPM images can display the layered structure of the blood vessels with higher contrast (Figure 7B). In terms of perilesional hemosiderosis, MPM can identify hemosiderin particles at a micron scale. H&E had higher specificity for the nuclear atypia. Table 2 overviewed the qMPM functions and promising clinical significance related to CCM histopathological features. The combination of Table 1 and 2 can serve as guidelines for pathologists and surgeons to train image-assisted diagnosis of qMPM. Our results suggested that qMPM could complement traditional histopathological diagnostic methods in the clinical workflows, which will make a more definitive intraoperative or postoperative CCM diagnosis.

## Discussion

The International League Against Epilepsy (ILAE) Commission has reported the predictors of CCM postoperative seizure freedom, including the small size of the CCM lesion, a lower preoperative seizure frequency, and removal of the surrounding hemosiderin rim 2. The extent of resection is the strongest but controversial predictor 3, 6-8, 28. The malformed blood vessels in the CCM lesion are prone to rupture and lead to microhemorrhage, and may eventually develop hyaline degeneration, collagen aging, gliosis, hemosiderin, and even focal cortical dysplasia in the adjacent tissue, therefore could make the diagnosis complicated 29. Moreover, hemosiderosis in the perilesional parenchyma has been proposed to induce epileptogenesis due to free iron and radicals generating a multitude of intracellular reactions 2, 30, 31. Therefore, the visualization of cavernous diseased vessels and hemosiderin-related deposition can assist neurosurgeons to determine the extent of resection more precisely.

MPM is the most suitable tool for imaging the histopathological features according to the spectral differences of endogenous molecules. Inspired by the resection requirements of CCM-associated epilepsy, we focused on two critical regions, the vascular pattern in the lesion and the hemosiderosis in the perilesional region. In this study, we firstly identified four vascular patterns using two-channel multiphoton imaging modality and then revealed three micron-scale hemosiderin-related products with three-channel multiphoton imaging modality. Following that, we developed three post-processing algorithms for CCM multiphoton images, combined as qMPM, to provide more effective guidance for resection. The first is a classifier that can automatically inform clinicians about vascular patterns based on SHG image features with 96.9 % AUC value, which provide a postoperative evaluation tool and will contribute to the examination of the association between different vascular patterns and the risk of CCM hemorrhage. The second is a quantitative visualization that can map different degrees of hemorrhage regions and cumulative hemosiderin onto fresh tissue like intraoperative fluorescence, and provide three quantitative hemosiderin-related parameters, which has the potential to guide neurosurgeons to perform label-free intraoperative resection more precisely. The third is deep-learned virtual staining. Virtual H&E and PPB stained images combined original staining information with unique MPM features to achieve higher histopathological diagnostic accuracy in blind analysis, validating the legibility of multiphoton images. In comparison to conventional MPM, qMPM solved the CCM diagnosis issues from the both dimensions of imaging capability expansion and customized algorithm development, provided MPM with more comprehensive quantitative information, as well as demonstrated the potential application value of qMPM in CCM resection.

Different resection strategies, such as pure lesionectomy, lesionectomy including hemosiderin rim, and extended resections, are critical to the prognosis of CCM-associated epilepsy. Most studies have reported significantly better outcome of CCM-associated epilepsy when the surrounding hemosiderin rim and gliosis were removed 1, 2, 5, 30-37. However, there is also some debate on the lesions and the outcomes of epilepsy 35, 38-40, and the duration of epilepsy is associated with multiple underlying factors (e.g., white matter volume loss or asymmetrical brain morphologic changes) 41-43. To be sure, the histopathological changes in CCM surrounding or remote tissue definitely play a significant role in the generation of seizures 1, 30, 32, 38, 44. In addition to hemosiderin and gliosis visualized in this study, MPM also has been shown to be capable of detecting secondary epileptogenic contributors that affected resection, such as neuronal damage, cortical dyslamination, and hippocampal sclerosis 23, 45-47. Hence, the proposed qMPM not only contributes to the histopathology-level resection of CCM-associated epilepsy, but will provide additional insights into the controversial discussion on the value of removing hemosiderin tissue for seizure outcomes.

However, it is also worth mentioning that the several practical considerations for clinical adoption of this approach. Firstly, although qMPM has the potential to assist clinicians for resection, microhemorrhage is not the only predictor that induces epilepsy. It is currently difficult to detect all epileptogenic zone using label-free optical imaging 33, 38, 40, because some remote epileptic foci and eloquent cortex need to be identified in combination with functional techniques, such as intraoperative electrocorticography (iECoG) or intraoperative MRI (iMRI) 33, 39. More importantly, if the hemosiderin-related lesion is involving or closer to eloquent regions, removal of hemosiderin is not recommended in order to prevent neurological deficits, even when epileptogenic foci have been identified by iECoG 31, 33. Therefore, qMPM can currently minimize the postoperative seizures rather than completely achieve seizure freedom. We are trying to collect more multicenter samples to provide stronger evidences for the prediction of the margins, and the robust of algorithmic models. On the other hand, the customized image algorithms are post-processing tool implemented in qMPM, which needs to acquire the images first and then process them. From bench to bedside, clinical qMPM need integrate the image processing algorithms into multiphoton imaging system with the heterogeneous parallel computing platform, resulting in a fully automatic and real-time intraoperative resection diagnosis.

As label-free MPM has progressed in clinical fundamental research, clinical limitations such as low intraoperative imaging resolution and insufficient imaging depth have emerged. Fortunately, these challenges have guided the advancement of multi-mode 48 and miniaturization 49, 50 multiphoton instruments, presenting a series of cutting-edge imaging technologies with multiple endogenous contrasts, smaller device size, and faster scanning speed. As a result, our preliminary results laid the foundation for the clinical requirements of CCM. The adoption of qMPM will promote the development of MPM diagnostic systems. With the iterative optimization of GRIN lens 51, photonic crystal fiber 52, laser source 53, as well as intelligent algorithm 54, future miniaturized hand-held quantitative multiphoton fiberscope will combine iMRI, iECoG, and functional navigation to assist neurosurgeons in tailoring resection of CCM lesions, most of the non-functional hemosiderin rim, and epileptogenic foci 33. This fusion of multimodal and multiscale features not only avoids damage to surrounding eloquent areas, but also has the greater potential to design a suitable approach and trajectory for precise resection 33, 37, 55, 56. In addition, the joint research of qMPM and clinical instruments could also develop a link between clinical needs and imaging researchers, so that qMPM can be applied in more disease diagnosis, and further enhance the translational potential of qMPM.

## Materials and methods

### Sample preparation

A total of 35 specimens were collected from 12 patients with CCM-related epilepsy. The imaging results and corresponding histological diagnosis were jointly reviewed by an imaging researcher (S.W.), a trained neuropathologist (X.W.), a neurosurgeon (S.S.), and a radiologist (R.L.). All patients either provided written informed consent or had an authorized representative consent on their behalf for tissue biopsy collection. All processes were approved by the Fujian Medical University Clinical Research Screening Committee for Studies Involving Human Subjects.

After tissue resection, the tissue sample was cut into serial sections of 10 μm thickness by a freezing microtome. For multiphoton microscopic imaging experiment, the section was perfused with a small amount of phosphate-buffered saline (PBS) to avoid tissue shrinkage, and placed a cover slip on the PBS-moistened specimens. The adjacent sections were stained with H&E, EVG, and PPB according to the standard protocol for the histopathological control experiment. These three different stainings were used to verify the multiphoton imaging results of CCM histopathological features, vascular structure, and hemosiderosis, respectively.

### MPM system

The MPM system mainly included a laser scanning microscope (Zeiss LSM 880 META, Jena, Germany) and an external mode-locked Ti: sapphire laser (140 fs, 80 MHz), tunable from 690 to 1064 nm (Chameleon Ultra, Coherent, Inc., Santa Clara, California). The MPM system schematic was presented in Figure S2. In our experiment, the optimal imaging excitation wavelength was 810 nm. The SHG/TPEF signals were generated on the sample using an average laser power of 30 mW. The emission signals were either spectrally separated by passing through a grating onto the 32-channel GaAsP photomultiplier tube (PMT) array detectors to obtain the TPEF signal and onto a flanking PMT detector to get the SHG signal. Additionally, the 32-channel GaAsP PMT array detectors (410-695 nm) also used to obtain the emission spectrum intensity. The spectral resolution was 9 nm.

To rapidly detect large-scale multiphoton images, two independent channels were set as follows: (i) SHG channel (395-415 nm, color-coded green) was used to visualize the vascular structures; (ii) TPEF channel (428-695 nm, color-coded red) was mainly used to visualize the cellular structures. To be more specific, we set up three independent channels to identify CCM histopathological details: (i) SHG channel (395-415 nm, color-coded green); (ii) TPEF-1 channel (428-570 nm, color-coded red) was mainly used to visualize NADH and FAD from cells; (iii) TPEF-2 channel (600-695 nm, color-coded cyan to white) was used to detect hemosiderin-related products. The large-scale multiphoton images were obtained by a Plan-Apochromat objective (10×/N.A.=0.45, Zeiss) for evaluating the tissue architecture. We acquired histopathological details at different scales by switching to a Plan-Apochromat objective (20×/N.A.=0.8, Zeiss) or zooming in on the region of interest (ROI). To obtain whole-slide multiphoton pathological images, the mosaic imaging of the sample was performed by transverse (xy) scanning of the motorized microscope stage (H1P2SLSM, Prior Scientific Instruments Ltd., Cambridge, UK). All frames (1024 × 1024 pixels, 12-bit pixel depth) were automatically recorded and stitched by Zeiss software, and the adjacent frames had 20% overlap. The acquisition time of a single frame (512 × 512 pixels) took 0.077 s by the bidirectional scanning mode.

### Classification of vascular patterns

A total of 142 features, including 8 morphologic features and 134 textural features, were extracted based on SHG images (*n* = 82) performed the collagen feature extraction algorithm 22, 23. The least-absolute shrinkage and selection operator (LASSO) logistic regression was used to select the most potential predictors from high-dimensional data. Subsequently, the 23 most instructive features, consist of 5 morphological features and 18 texture features, were selected according to the optimal value of λ (0.006579) determined by five-time cross-validations. The SVM classifier was established to automatically discriminate the vascular patterns utilizing the 23 features. The leave-one-out cross-validation method was used to prevent overfitting and to evaluate the generalization ability of the model. We used a receiver operating characteristic (ROC) curve as the ultimate evaluation criterion of the classifier's performance.

### Quantitative visualization of hemorrhage regions

We calculated three hemosiderin-related parameters in a three-channel MPM image, including serious hemorrhage ratio, hemorrhage extent and cumulative hemosiderosis level, to quantitatively visualize the hemorrhage-related areas. The area of SHG signal (S*_SHG_*), TPEF-1 signal (S*_TPEF-1_*), and TPEF-2 signal (S*_TPEF-2_*) were obtained by summing up the pixels of each channel. The intensity of TPEF-2 signal (I*_TPEF-2_*) was quantified by calculating the total intensity of pixels in TPEF-2 channel. The above parameters are given by


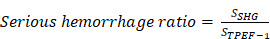

(1)


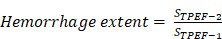

(2)


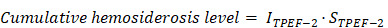

(3)

The hemorrhage center area was mapped in color-coded red according to the value of serious hemorrhage ratio. The hemorrhage edge area was visualized based on the difference set of hemorrhage extent and serious hemorrhage ratio, mapped in color-coded yellow. The perihemorrhagic hemosiderosis area was the difference set of cumulative hemosiderosis level and hemorrhage extent, mapped in color-coded blue. Then, three areas were visualized in form of a heatmap onto the fresh tissue. The above processes were implemented by Python 3.9.6 (Python Software Foundation). Finally, the neurosurgeon (S.S.) and neuropathologist (X.W.) verified the visualization results.

### CCM-stainGAN

CCM-stainGAN can transform label-free MPM images to the corresponding H&E or PPB histopathological stained images without pixel-level registered MPM-staining training pairs based on the CycleGAN framework 57. CycleGAN has the capacity to learn the transformation from the MPM images to the stained images, but shows weak constraint in the complex histopathological domains. Therefore, CCM-stainGAN additionally introduced the deep feature consistency and tissue component consistency for more reliable transformation task of complex histopathological features in CCM (Fig. 6A). The tissue component consistency was mainly reflected in the classification of tissue components, such as various vascular patterns and hemosiderin-related products, which was crucial information for correct transformation of the different tissue component. A classifier was built independently from the generator, which adds classification constraint to the transformation of images. The tissue component consistency requires the component outputs of classifier to be identical as well for the corresponding domains. The deep feature consistency contained de-staining feature constraint and saliency content constraint 58, which was used to achieve high-level transformation of the same tissue component. More details can be found in Note S1 and Note S2 in Supplementary Material. Furthermore, an ablation study was performed to determine the contribution of loss functions to the performance of CCM-stainGAN (Figure S3 and Figure S4).

The generator architecture of CCM-stainGAN was modified based on the skip connection and residual net (Figure S5). The generator concatenated the encoder and decoder by using the skip connection to improve structure details and utilized residual block to prevent the gradients vanish in deep network. For the discriminator, we adopted the PatchGAN classifier, which encouraged high-frequency details of the image. In the training process, the network parameters were optimized by the Adam optimizer, and the model was trained by end-to-end backpropagation. The learning rate is initially set at 0.0002, and the exponential decay rate is 0.999. The above processes used Python 3.6 based on the open-source deep-learning library PyTorch on a single NVIDIA RTX 3090 with 24 G memory for training and testing.

### Statistical analysis

Statistical analyses were performed using GraphPad Prism software (version 9.0.0, GraphPad Software). To compare three or more groups, a one-way analysis of variance (ANOVA) was utilized, followed by Tukey's multiple comparisons test. If the normality test failed, Kruskal-Wallis test and Dunn's multiple comparisons test were used. The significance level is displayed as asterisks, and the value *P* < 0.05 is considered to be statistically significant (^*^P < 0.05, ^**^P < 0.01, ^***^P < 0.001, ^****^P < 0.0001; NS, not significant).

### Blinded analysis

We subjected datasets from 10 specimens to a blind diagnostic analysis. MPM images were randomly divided into 'training set' and 'validation set' according to the ratio of 2:8. The 'training set' consisted of 28 MPM images (5 normal vessels, 3 gray matter, 5 vascular malformation, 6 hyaline degeneration, 4 aging vessels, and 5 hemosiderin) and corresponding H&E-stained images. The remaining MPM images, H&E-stained images, and virtual-stained images (a total of 333 images) were assigned as the 'validation set'. Three neuropathologists (L.Z., C.H., and X.W.) observed the 'training set' to learn and familiarize with the optical characteristics on MPM images. The training process took less than 30 min. Afterward, the trained neuropathologists were shown the blinded MPM images in the 'validation set', and then classified categories of diagnostic features. One week later, the same neuropathologists diagnosed the corresponding H&E-stained images. Another week later, they diagnosed the virtual-stained images. Lastly, the results were compared to the blind codes for diagnostic accuracies and further analysis.

## Supplementary Material

Supplementary figures, tables, material.

## Figures and Tables

**Table 1:** CCM histopathological features observed in MPM and the corresponding H&E-stained images.

Histopathological features	MPM	H&E
**Normal vessels**	Distinct three-layered vascular structures, consisting collagen fiber (SHG), smooth muscle (TPEF), and elastic fibers (TPEF)	Vascular layer structures (pink color), endothelial cells (blue color)
**Vascular malformation**	Multiple dilated blood-filled caverns with little or no intervening brain parenchyma (TPEF). Vascular wall appears elongated collagen fiber (SHG) without the smooth muscle and elastic fiber	Multiple dilated blood-filled caverns with little or no intervening brain parenchyma
**Hyaline degeneration**	Homogeneous translucent regions (SHG) and dense fibrillar collagen deposition (TPEF) in the vascular wall, displaying unobvious vascular cavity	Dense and homogeneous fibrillar collagen without obvious vascular cavity
**Vascular collagen aging**	Twisted vascular wall (both SHG and TPEF)	Twisted vascular wall (blue color)
**Gliosis**	Higher cell density (TPEF) associated with hemosiderosis or vascular degeneration (TPEF or SHG)	Proliferation or hypertrophy of glial cells
**Hemosiderin-storing macrophages**	Dark nucleus surrounded by granules (strong TPEF)	Foamed shape macrophages (need confirmed by PPB)
**Hemosiderin**	Granular deposition (both strong SHG and TPEF)	Granular deposition (some appear yellow or brown color, need confirmed by PPB)

CCM: Cerebral cavernous malformation; MPM: multiphoton microscopy; H&E: hematoxylin-eosin; SHG: second harmonic generation; TPEF: two-photon excited fluorescence; PPB: Perls Prussian Blue.

**Table 2:** The guidelines for qMPM histopathological diagnosis of CCM.

Histopathological features	Vascular structure	Cellular structure	Gliosis	Hemosiderin-laden macrophages	Hemosiderin
**Modality/target substances**	SHG/collagen fiber; TPEF-1/smooth muscle; SHG, TPEF-1/elastic fiber	TPEF-1/ hemoglobin, NADH, FAD, porphyrin	TPEF-1/ NADH, FAD	TPEF-2/ hemoglobin, NADH, FAD	SHG/ferritin; TPEF-2/iron-binding complexes
**Excitation wavelength (nm)**	790-860 (810, optimum)	770-890 (810, optimum)			
**Customized post-processing algorithms**	The classifier of vascular patterns				
	Quantitative visualization of hemorrhage regions, deep-learned virtual staining (CCM-stainGAN)				
**Observed events**	Structural changes of vascular wall, vascular hyaline degeneration, collagen aging	Cell morphologic changes, metabolic changes	Visualization of the perilesional hemorrhage regions, visualization of microhemorrhages sites, hemosiderin-related parameters		
**Potential clinical applications**	Postoperative prognosis evaluation	Histopathological diagnosis	Intraoperative resection guidance, CCM-related seizures analysis, surgical endoscopy		
**Imaging advantages**	High specificity and sensitivity	Single-cell resolution	Multiple molecular signal	Micron-scale hemosiderin	Strong autofluorescence signal
**Imaging limitations**	Microvascular signal is slightly weak	Lack of intranuclear details may impair ability to detect small morphological changes in glial cells		Smaller iron deposits might be confused with impurities	Photodamage risks caused by long time excitation
**Histopathological staining methods**	Masson/EVG	H&E	GFAP	PPB	PPB

CCM: Cerebral cavernous malformation; qMPM: quantitative multiphoton microscopy; SHG: second harmonic generation; TPEF: two-photon excited fluorescence; NADH: nicotinamide adenine dinucleotide; FAD: flavin adenine dinucleotide; H&E: hematoxylin-eosin; EVG: Elastin van Gieson; PPB: Perls Prussian Blue; GFAP: glial fibrillary acidic protein.

## Abbreviations

CCM: cerebral cavernous malformation
qMPM: quantitative multiphoton microscopy
MRI: magnetic resonance imaging
MPM: multiphoton microscopy
SHG: second harmonic generation
TPEF: two-photon excited fluorescence
H&E: hematoxylin-eosin
EvG: Elastic van Gieson
NADH: nicotinamide adenine dinucleotide
FAD: flavin adenine dinucleotide
AUC: area under curve
LASSO: least absolute shrinkage and selection operator
SVM: linear support vector machine
SHR: serious hemorrhage ratio
HRE: hemorrhage extent
CHSL: cumulative hemosiderosis level
HRCA: hemorrhage center area
HREA: hemorrhage edge area
PHSA: perihemorrhagic hemosiderosis area
HNA: histologically normal area
ANOVA: one-way analysis of variance
RBCs: red blood cells
PPB: Perls Prussian Blue
FOV: field of views
CCM-stainGAN: CCM-oriented virtual staining generative adversarial network
ILAE: International League Against Epilepsy
PBS: phosphate-buffered saline
PMT: photomultiplier tube
ROC: receiver operating characteristic
iECoG: intraoperative electrocorticography
iMRI: intraoperative MRI
