# Supplementary material for: Resection-inspired histopathological diagnosis of cerebral cavernous malformations using quantitative multiphoton microscopy
Source: Theranostics. 2022 Sep 11;12(15):6595–610. doi: 10.7150/thno.77532 (PMC9516234; doi:10.7150/thno.77532)
Supplement: Supplementary file 1 — Supplementary figures, tables, material. [file thnov12p6595s1.pdf]

Supplementary Information

Resection-inspired histopathological diagnosis of cerebral cavernous malformations using quantitative multiphoton microscopy

Shu Wang[1, 2, #], Yueying Li[1, #], Yixuan Xu[1, #], Shiwei Song[3], Ruolan Lin[4], Shuoyu Xu[5], Xingxin Huang[2], Limei Zheng[6], Chengcong Hu[6], Xinquan Sun[1], Feng Huang[1, *], Xingfu Wang[6, *], Jianxin Chen[2, *]

[1]College of Mechanical Engineering and Automation, Fuzhou University, Fuzhou 350108, China
[2]Key Laboratory of OptoElectronic Science and Technology for Medicine of Ministry of Education, Fujian Provincial Key Laboratory of Photonics Technology, Fujian Normal University, Fuzhou 350007, China
[3]Department of Neurosurgery, Fujian Medical University Union Hospital, Fuzhou 350001, China
[4]Department of Radiology, Fujian Medical University Union Hospital, Fuzhou 350001, China
[5]Department of General Surgery, Nanfang Hospital, Southern Medical University, Guangzhou 510515, China
[6]Department of Pathology, the First Affiliated Hospital of Fujian Medical University, Fuzhou 350004, China
[#]*These authors contributed equally to this work.*
[*]*Correspondence: Jianxin Chen (chenjianxin@fjnu.edu.cn); Xingfu Wang (wang_xfu@126.com); Feng Huang (huangf@fzu.edu.cn).*

This PDF file includes:

Figure S1. Quantitative visualization of the hemorrhage-related region (the first position in Figure 4B).
Figure S2. The MPM system schematic.
Figure S3. Ablation study of virtual H&E staining.
Figure S4. Ablation study of virtual PPB staining.
Figure S5. CCM-stainGAN architecture.
Table S1. The collagen features extracted using the algorithm.
Table S2. Comparative CCM diagnosis results among MPM, H&E and virtual staining of blinded analysis.
Note S1. Network architecture and training details.
Note S2. Loss function for network.

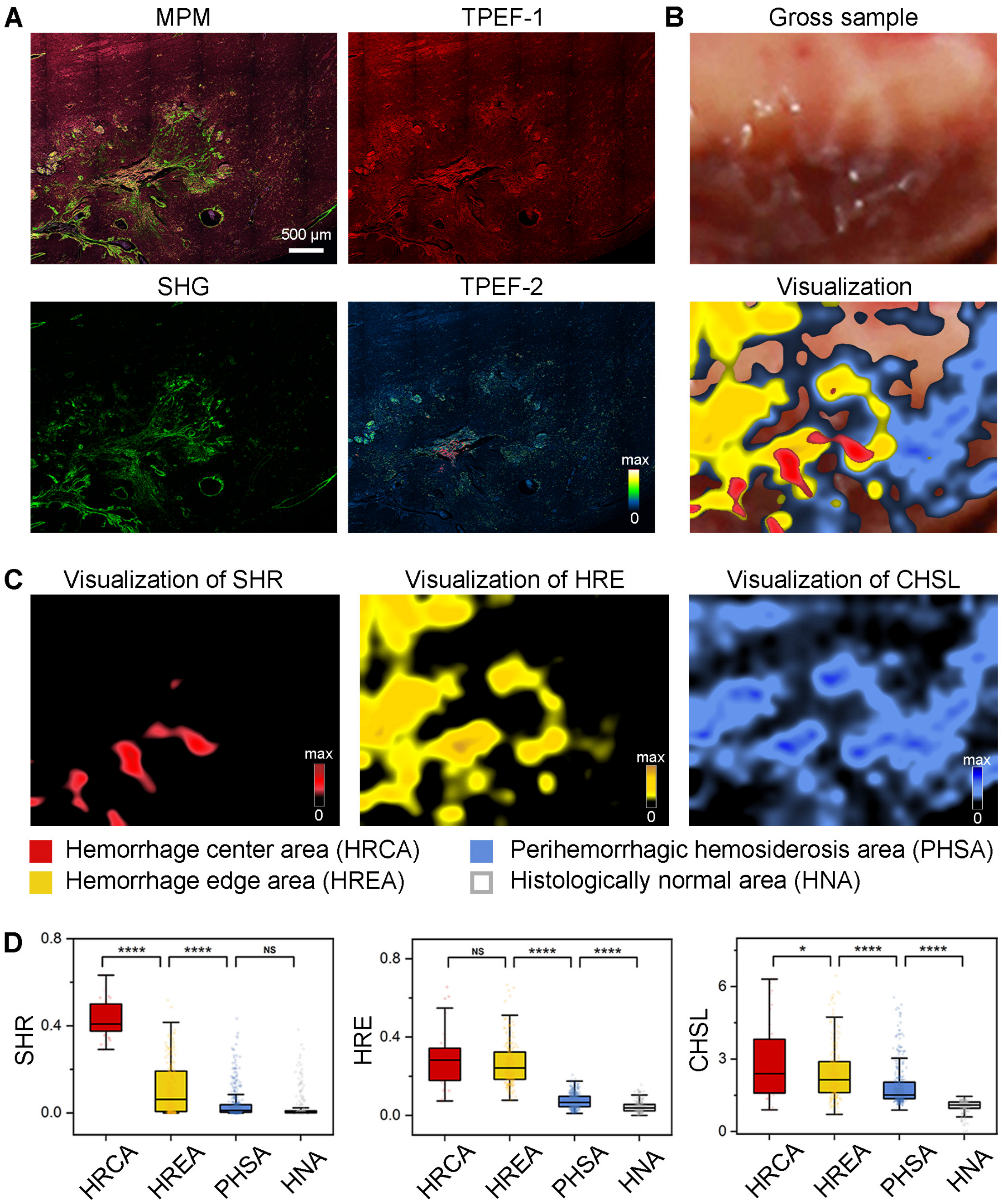

Figure S1. Quantitative visualization of the hemorrhage-related region (the first position in Figure 4B). (A) The MPM images. **(B)** The visualization of hemorrhage-related areas compared to gross sample, which including hemorrhage center area (HRCA, color-coded red), hemorrhage edge area (HREA, color-coded yellow), and perihemorrhagic hemosiderosis area (PHSA, color-coded blue). The uncovered area was the histologically normal area (HNA). **(C)** The visualization of hemosiderin-related parameters (i.e., serious hemorrhage ratio (SHR), hemorrhage extent (HRE), and cumulative hemosiderosis level (CHSL)), which provided the support for the visualization of four hemorrhage-related areas. **(D)** The quantitative calculation of SHR, HRE, and CHSL in the four areas ($n_{HRCA} = 35$, $n_{HREA} = 246$, $n_{PHSA} = 433$, $n_{HNA} = 245$, n depends on the size of the area, field of view (FOV) = 816 μm × 816 μm). The parameter values showed a decrease with the expansion of resection-guided areas. Centerlines, medians; limits, 75 and 25%; whiskers, maximum and minimum.

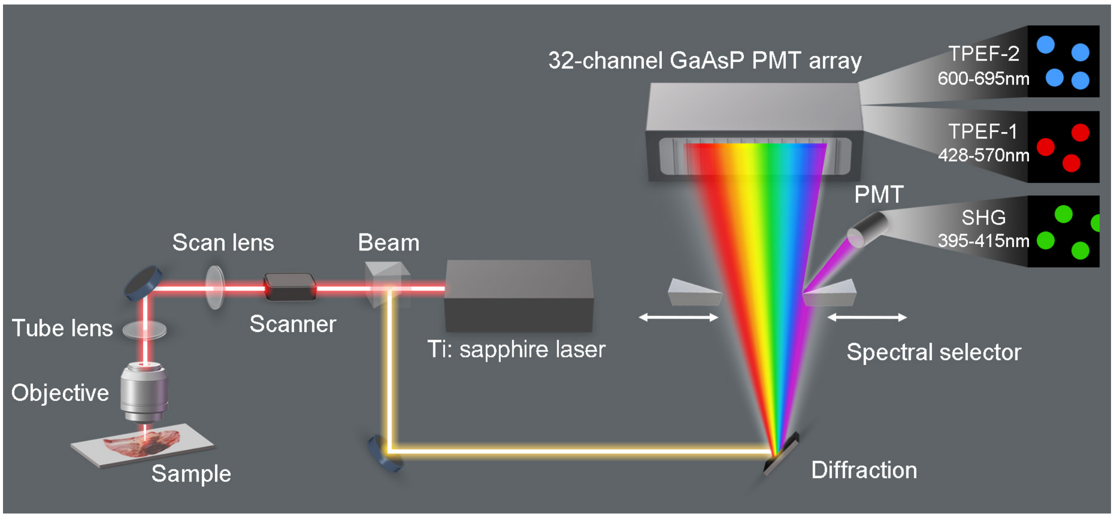

Figure S2. The MPM system schematic. The laser beam is focused onto the specimen through an objective. The light emitted at the focal plane is directed via an XY scanner onto a dichroic beam splitter, which separates the emissions from the excitation light. In the detection module, the emitted fluorescence is either spectrally separated by passing through a grating onto the 32-channel GaAsP PMT array and one additional PMT. For multi-channel detection, the spectral selector with an adjustable mechanical slit is used to select a band of wavelengths from the spectrum.

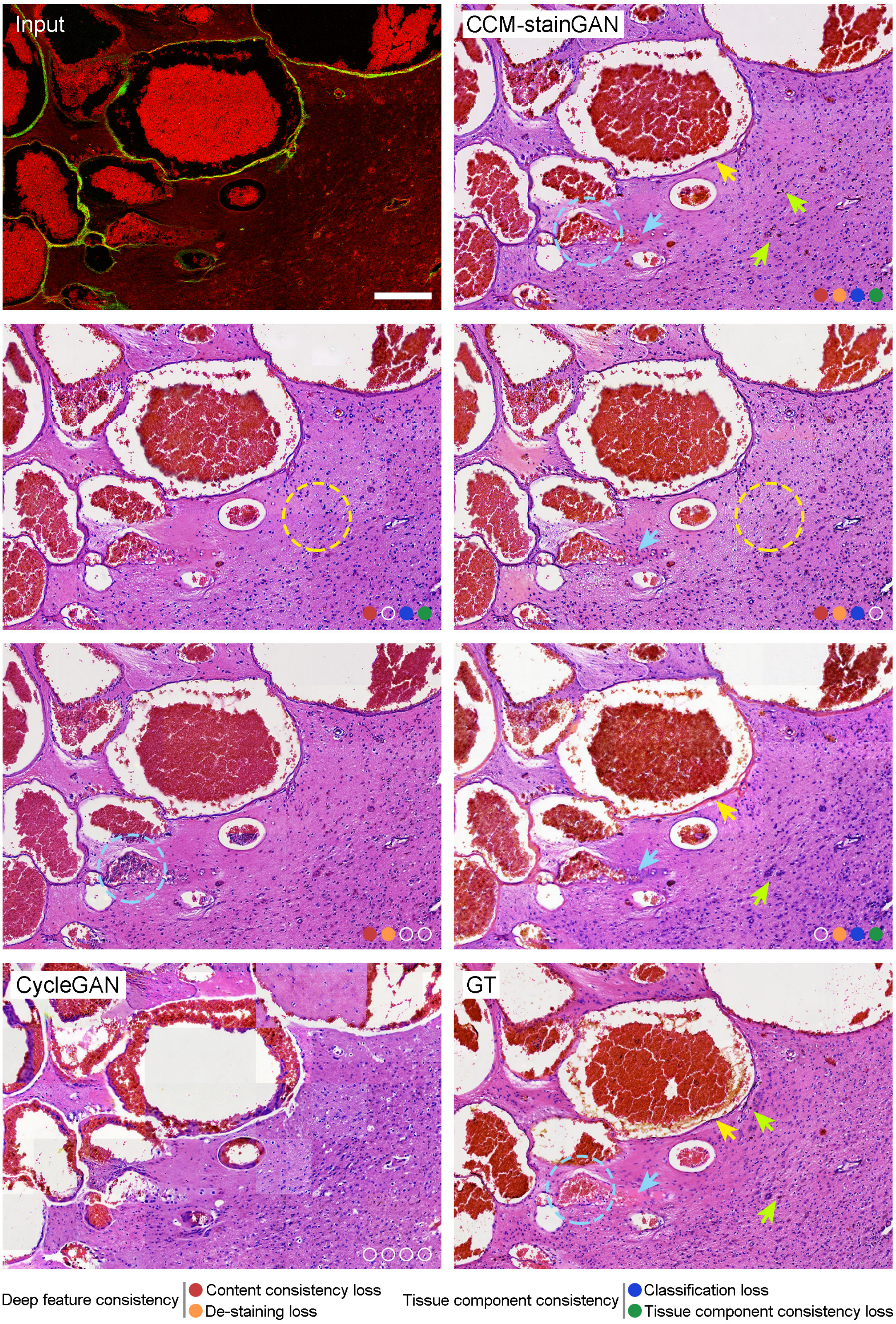

Figure S3. Ablation study of virtual H&E staining. CCM-stainGAN with all loss functions performed a higher capacity for histopathological fidelity. Specifically, with the tissue component consistency loss or de-staining loss, the stitching artifacts (all patches were stitched with 50% overlap) of CCM-stainGAN was significantly improved (yellow dashed circles). Some details, such as vascular wall (yellow arrows), black granular hemosiderin (green arrows) and cell density (blue

arrows), were susceptible to tissue component consistency loss and content consistency loss. The transformation of CCM-stainGAN, benefited from the classification loss and the tissue component consistency loss, can minimize the misidentification (blue dashed circles). Scale bar, 200 μm.

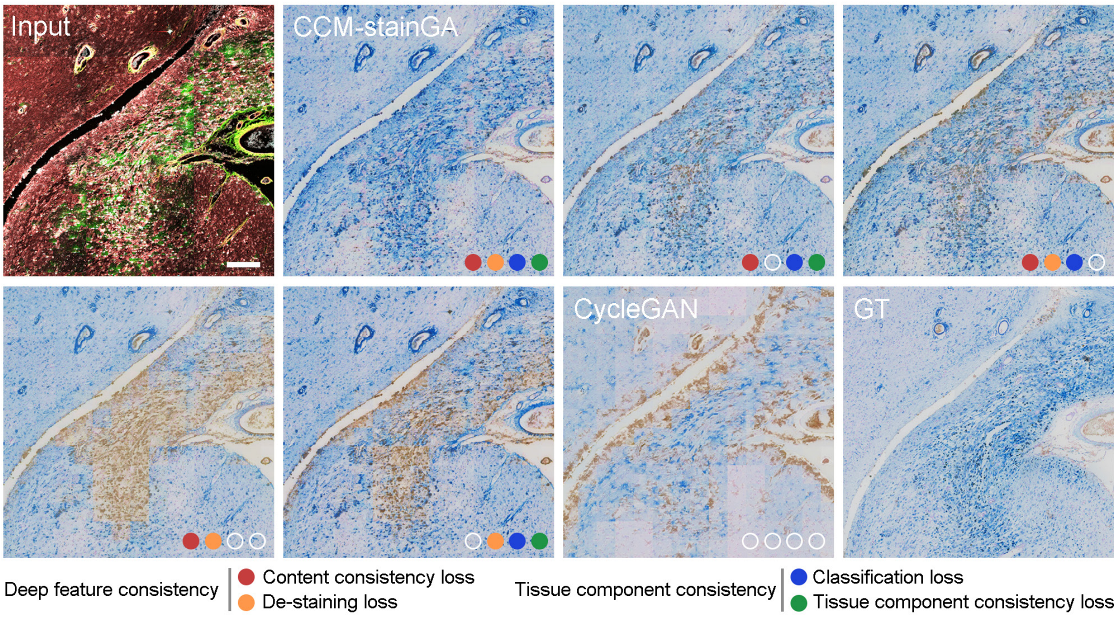

Figure S4. Ablation study of virtual PPB staining. Introducing the de-staining loss or tissue consistency loss, the CCM-stainGAN can accurately transform the tissue components, particularly exceled in the recovery of blue-black granular hemosiderin. With the classification loss or content consistency loss, the stitching artifacts almost disappeared in transformations. CCM-stainGAN with all loss functions performed a higher capacity for histopathological fidelity than CycleGAN. Scale bar, 200 μm.

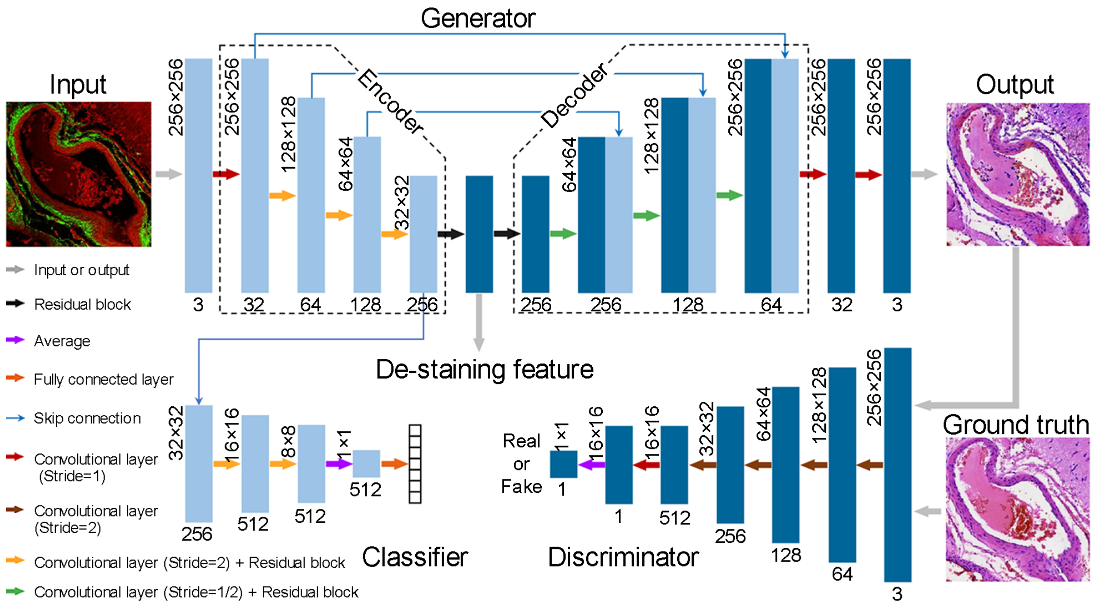

Figure S5. CCM-stainGAN architecture. The generator section is used to transform the MPM images into stained images, which consists of the encoder and decoder for extracting features and re-staining. Skip connections are used to pass the data between the same level encoder and decoder. The generator shares information with the classifier, which is trained by using the training and labeled data. The discriminator utilizes a PatchGAN classifier that penalizes small patches to encourage the details.

Table S1. The collagen features extracted using the algorithm.

	No.	Feature descriptions
**Morphological features**	1-2	Collagen fiber percentage area, number of collagen fibers
	3-4	Collagen fiber length, collagen fiber width
	5-6	Collagen fiber straightness, collagen cross-link density
	7-8	Collagen cross-linking volume, collagen orientation
**Texture features**	9-14	Mean, variance, skewness, kurtosis, energy, entropy
	15-94	Contrast, correlation, energy and homogeneity from the gray-level co-occurrence matrix given five different pixel distances with four different directions
	95-142	Mean and variance of the convolution over the image with the Gabor filter at four scales with six orientations

Table S2. Comparative CCM diagnosis results among MPM, H&E and virtual staining of blinded analysis.

Diagnosis	Modality	Pathologist 1		Pathologist 2		Pathologist 3		Overall Accuracy (%)
		Correct	Incorrect	Correct	Incorrect	Correct	Incorrect	
Normal vessels	MPM	11	0	11	0	10	1	96.97
	Virtual	11	0	9	2	11	0	93.94
	H&E	10	1	10	1	9	2	87.88
Vascular malformation	MPM	20	1	21	0	21	0	98.41
	Virtual	21	0	21	0	21	0	100
	H&E	21	0	21	0	20	1	98.41
Hyaline degeneration	MPM	19	3	22	0	22	0	95.45
	Virtual	21	1	22	0	22	0	98.48
	H&E	21	1	21	1	22	0	96.97
Vascular collagen aging	MPM	7	4	8	3	9	2	72.73
	Virtual	11	0	11	0	11	0	100
	H&E	11	0	11	0	9	2	93.94
Hemosiderin	MPM	36	1	35	2	36	1	96.40
	Virtual	37	0	37	0	36	1	99.10
	H&E	36	1	36	1	35	2	96.40
Gray matter	MPM	9	0	9	0	9	0	100
	Virtual	9	0	9	0	9	0	100
	H&E	9	0	9	0	9	0	100
Total	MPM	102	9	106	5	107	4	94.59
	Virtual	110	1	109	2	110	1	98.80
	H&E	108	3	108	3x	104	7	96.10
Accuracy (%)	MPM	91.89		95.50		96.40		
	Virtual	99.1		98.2		99.1		
	H&E	97.30		97.30		93.69		

Note S1. Network architecture and training details

For the architecture of CCM-stainGAN, we designed the generator and classifier based on the residual block and the skip connection of the U-net. The first three layers of the generator extract abstract features by downsampling layers with residual blocks. A downsampling path consists of three individual steps, which maps a feature map x_k into another feature map x_{k+1}:

$$x_{k+1} = \mathrm{Res}[\mathrm{LReLU}\{\mathrm{Conv}[x_k]\}] \quad (1)$$

Where $\mathrm{Conv}\{\cdot\}$ is the convolutional layer that includes the bias terms, and the stride = 2 to reduce the size of the images while increasing the number of the channels. $\mathrm{LReLU}\{\cdot\}$ is the nonlinear activation function (leaky rectified linear unit) for the network, which defined as:

$$\mathrm{LReLU}=\begin{cases} x, & if\, x\geq 0 \\ ax, & if\, x<0 \end{cases} \quad (2)$$

in this paper, $a = 0.02$. And the $\mathrm{Res}\{\cdot\}$ represents the residual block, which can alleviate the problem of vanishing gradients in deep network.

$$\mathrm{Res}{=}x + \mathrm{LReLU}\{\mathrm{Conv}[\mathrm{LReLU}\{\mathrm{Conv}[x]\}]\} \quad (3)$$

N residual blocks follow the three downsamplings to adapt to the complex task. De-staining feature at the n/2 residual blocks (Figure S5) is one of the outputs of the network, which should keep consistency with the corresponding cycle de-staining feature. Three upsampling layers follow the output of n residual blocks to re-stain the image. An upsampling layer consists of four steps, each layer concatenates the output of the same layer in the encoder, which maps a feature map y_k into feature map y_{k+1}:

$$y_{k+1} = \mathrm{Res}\{\mathrm{LReLU}[\mathrm{Conv}k_2\{\mathrm{Concat}[x_{k+1},\mathrm{LReLU}\{\mathrm{Conv}k_1[y_k]\}]\}]\} \quad (4)$$

where $\mathrm{Concat}[\cdot]$ is the concatenation between two feature maps that merges the number of channels. $\mathrm{Conv}k_1$ is the upsampling layer, which increases the size of the image while reducing the number of channels, $\mathrm{Conv}k_2$ denotes the convolutional layer that reduces the number of channels of the merged feature maps.

The classifier is independent of the generator but shares part of the network structure with the generator. The classifier follows the three downsamplings of the generator, which continues to downsample two times before average pooling. Finally, the classification is output through the fully connected layer (Figure S5). For the discriminator, we adopt the PatchGAN classifier (Figure S5) that penalizes small patches to encourage the details.

In the training dataset, the MPM images, H&E, and PPB images were randomly cropped into 256×256 patches. For the two-channel MPM images to H&E images phase, the total number of patches were 18021 and 17409 respectively. The number of three-channel and PPB images patches were 9462 and 9989 respectively. During the test process, we partitioned the large image into multiple tiles with 50% overlap and stitched the predicted tiles together, which reduced the artifact of the image by cutting the boundary (half of the overlap). The percentage of overlap determines

the number of tiles, which further changes the transformation time of the whole slide images. We trained the CCM-stainGAN about 400000 iterations with 48 h consumed on a single NVIDIA GEFORCE RTX 3090 GPU (24 GB memory). After training, CCM-stainGAN took 9.6 ms to transform a 256×256 MPM tile and 480 s to obtain a whole slide image.

Note S2. Loss function for network

For the generation phase, we utilized the original cycle consistency loss and adversarial loss of CycleGAN framework[1] to prevent pattern collapse, which defined as:

$$L_{cycle}(G,F) = \mathrm{E}_x[\|F(G(x)) - x\|_1] + \mathrm{E}_y[\|G(F(y)) - y\|_1] \tag{5}$$

$$\begin{aligned} L_{adv}(G,D_Y) &= \mathrm{E}_y[\log D_Y(y)] + \mathrm{E}_x[\log(1 - D_Y(G(x)))] \\ L_{adv}(F,D_X) &= \mathrm{E}_x[\log D_X(x)] + \mathrm{E}_y[\log(1 - D_X(G(y)))] \end{aligned} \tag{6}$$

where G and F respectively represent the forward generator and backward generator. D_X and D_Y are the discriminators of domains X and Y .

The tissue components are crucial information for histopathological virtual staining. For a more realistic and stable transformation, we built a classifier[2], which shares part of the network structure with the generator. The classification loss function is defined as:

$$L_{classification}(C_X, C_Y) = \mathrm{E}_x[\ell(C_X(x), c_x)] + \mathrm{E}_y[\ell(C_Y(y), c_y)] \tag{7}$$

Where $\ell(\cdot)$ is the Softmax Entropy Loss. C_X and C_Y are the vascular patterns classifiers of domains X and Y . The x , y are the samples and c_x , c_y are the correct labels from domains X and Y .

During the cycle process, the outputs of the classifiers should be identical as well. Thus, the tissue component consistency loss is defined with the L1-norm:

$$L_{component}(G,F,C_X,C_Y) = \mathrm{E}_x[\|C_X(x) - C_Y(G(x))\|_1] + \mathrm{E}_y[\|C_Y(y) - C_X(F(y))\|_1] \tag{8}$$

Content-preserving transformation was proposed to guide the transformation for optical microscopy[3]. The histopathological content of the image is separated from the background by using a threshold segmentation. Subsequently, the content is preserved for cross-domain transformation through the content loss, which is defined as:

$$L_{content}(G,F) = \mathrm{E}_x[\|T_\delta(x) - T_\varepsilon(G(x))\|_1] + \mathrm{E}_y[\|T_\varepsilon(y) - T_\delta(F(y))\|_1] \tag{9}$$

$$\begin{aligned} T_\delta(x) &= sigmoid[100(x - \delta)] \\ T_\varepsilon(y) &= 1 - sigmoid[100(y - \varepsilon)] \end{aligned} \tag{10}$$

where T_δ and T_ε are segmentation functions parameterized by thresholds δ and ε respectively, and the thresholds are manually selected for the network. The image content is mapped to a value of 1 and the background is to a value of 0, which is easy for backpropagation after the sigmoid function mapping.

To achieve high-level virtual staining, we modeled virtual staining process in two parts: de-staining and re-staining. In the de-staining process, the encoder extracts significant semantic information. For different corresponding domains, the encoder extracts the same feature after the de-staining phase[4]. The de-staining loss is aligned using L1-norm:

$$L_{de\text{-}staining}(G,F) = \mathrm{E}_x[\|G_{en}(x) - F_{en}(G(x))\|_1] + \mathrm{E}_y[\|F_{en}(y) - G_{en}(F(y))\|_1] \tag{11}$$

Where G_{en} and F_{en} represent the encoders of the generator.

Finally, the full loss function of CCM-stainGAN is formulated as follows:

$$
\begin{aligned}
& L(G,F,D_X,D_Y,C_X,C_Y) \\
& \quad = L_{adv}(G,D_Y) + L_{adv}(F,D_X) + \alpha L_{cycle}(G,F) \\
& \quad + \beta L_{de-stain}(G,F) + \gamma L_{content}(G,F) + L_{class}(C_X,C_Y) \\
& \quad + L_{ts}(C_X,C_Y)
\end{aligned} \tag{12}
$$

where α , β , γ are hyper-parameters, and α , β are empirically set 5 and 2. The γ is exponentially decay to a value of 0, which only constraints the early phase of the training.

Supplementary references

1. Zhu J, Park T, Isola P, Efros AA. Unpaired Image-to-Image Translation Using Cycle-Consistent Adversarial Networks. IEEE International Conference on Computer Vision (ICCV); 2017. p. 2242-51.
2. Sankaranarayanan S, Balaji Y, Castillo CD, Chellappa R. Generate to Adapt: Aligning Domains Using Generative Adversarial Networks. IEEE/CVF Conference on Computer Vision and Pattern Recognition; 2018. p. 8503-12.
3. Li X, Zhang G, Qiao H, Bao F, Deng Y, Wu J, et al. Unsupervised content-preserving transformation for optical microscopy. Light: Sci Appl. 2021; 10: 44.
4. Lahiani A, Navab N, Albarqouni S, Klaiman E. Perceptual Embedding Consistency for Seamless Reconstruction of Tilewise Style Transfer. Medical Image Computing and Computer Assisted Intervention (MICCAI); 2019. p. 568–76.
